# Localised effects of sparse natural tree colonisation on soil physicochemical properties

**DOI:** 10.1007/s00468-026-02789-0

**Published:** 2026-06-02

**Authors:** Naomi C. Housego, Thomas C. Parker, Lorna E. Street, Elena I. Vanguelova, Ruth J. Mitchell

**Affiliations:** 1 The James Hutton Institute, Aberdeen, UK; 2 School of GeoSciences, University of Edinburgh, Edinburgh, UK; 3 Forest Research, Alice Holt, Farnham, UK

**Keywords:** Heather moorland, Natural colonisation, Organo-mineral soils, Soil decomposition, Soil moisture, Soil pH

## Abstract

Tree establishment via natural colonisation is increasing globally, due to treeline expansion under climate change, and changing land management practices, such as reduced grazing pressure, land abandonment, and initiatives to increase tree cover such as restoration and rewilding. Natural colonisation of organo-mineral soils at high densities is associated with altered soil physicochemical properties, with consequences for ecosystem services such as soil carbon stocks, biodiversity, and hydrology. But whether sparse natural colonisation of organo-mineral soils has similar effects on soil physicochemical properties to dense natural colonisation is not known. To investigate the effects of sparse natural colonisation on organo-mineral soils, we measured soil physicochemical properties at increasing distances up to 8 metres from single, native (*Pinus sylvestris* L. or *Betula* spp.) naturally colonised trees in Cairngorms, Scotland, UK. Distance from single, native, naturally colonised trees was associated with increased soil moisture and decreased carbon-to-nitrogen and carbon-to-phosphorus ratios, but not associated with changes in bulk density, decomposition rates, or pH, in the organic horizon of organo-mineral soils. Sparse natural colonisation (in this study, 43 trees ha^− 1^) of organo-mineral soils has some, localised effects on the physicochemical properties of the organic horizon, which might affect biodiversity and hydrology in patches around trees. These effects did not extend beyond 2 metres distance from the trees. This suggests that early, sparse tree colonisation has minimal impacts on soil physicochemical properties at the ecosystem scale.

## Introduction

Natural tree colonisation – where trees establish from parent trees via seedfall and distribution without direct human intervention in locations not previously or recently forested – is increasing globally (Harsch et al. 2009). This increase in natural colonisation is occurring because of altitudinal and latitudinal treeline expansions under climate change, changing management approaches such as reduced grazing pressure and land abandonment, and initiatives that aim to restore forests and/or rewild landscapes (Song et al. 2018). Natural colonisation offers a cost-effective approach to increasing tree cover over large areas, where tree planting would not be affordable nor feasible (Crouzeilles et al. 2020).

Tree colonisation has been shown to alter soil physicochemical properties such as C: N, C: P, decomposition rates, pH, and soil moisture (e.g. Miles 1981; Behera and Sahani 2003; Acuña-Míguez et al. 2024). Increased C: N and C: P ratios are associated with tree planting across many ecosystems (Shi et al. 2016) and biomes (Zarafshar et al. 2023) and can occur if the C: N of tree litter inputs exceeds that of the vegetation that they are replacing (Vesterdal et al. 2008). Additionally, selective ‘N-mining’ by root-associated microbial communities, whereby N is extracted from soil organic matter at a higher rate than the C is mineralised can increase the soil C: N ratio (Hicks et al. 2020; Clemmensen et al. 2021). Tree establishment could also be linked with increased soil decomposition rates under trees, which could decrease the C: N ratio because free-living soil organisms require soil C for metabolism (Berg 2000; Alberti et al. 2015; Ostrowska and Porębska 2015; Blanco et al. 2023). Cellulose and lignin are major components of plant litter, thus form major components of the soil litter and humus layers (Štursová et al. 2012; Rahman et al. 2013). Their decomposition rates are affected by changes in soil microbial activity and community composition, pH, and moisture (Donnelly et al. 1990) that might occur following tree establishment. Tree establishment alters soil pH because trees take up different amounts of elements from the soil and have litter of a different chemical composition compared to the previous vegetation community (Finzi et al. 1998; van Meeteren et al. 2007). Tree establishment can alter soil moisture; canopy interception and transpiration can decrease soil moisture beneath the tree and within its rooting zone. Decreased soil moisture can also drive increased bulk density, as the loss of moisture causes the soil structure to collapse from the decreased support (Minkkinen and Laine 1998). Changes to soil moisture following tree establishment can also affect soil C, N, and P, as the release of these during decomposition is positively mediated by soil moisture (van Meeteren et al. 2007). Soil microbial activity and community composition, and therefore decomposition rates, are also affected by soil moisture changes following tree establishment (Nielsen et al. 2008; Weil and Brady 2016).

Previous work on the impacts of tree colonisation on soil physicochemical properties examined dense colonisation or dense tree planting. Far less is known about sparse natural colonisation, where the soil retains characteristics of the previous land use. Sparse natural colonisation (in this study, 43 trees ha^− 1^) reflects early stages of colonisation (which diffes from regeneration in that tree establishment occurs in locations not previously or recently forested), unfenced colonisation experiencing high grazing pressures, and/or colonisation of soils that are not experiencing strong influences of a tree on their soil physicochemical properties.

**Fig. 1:**
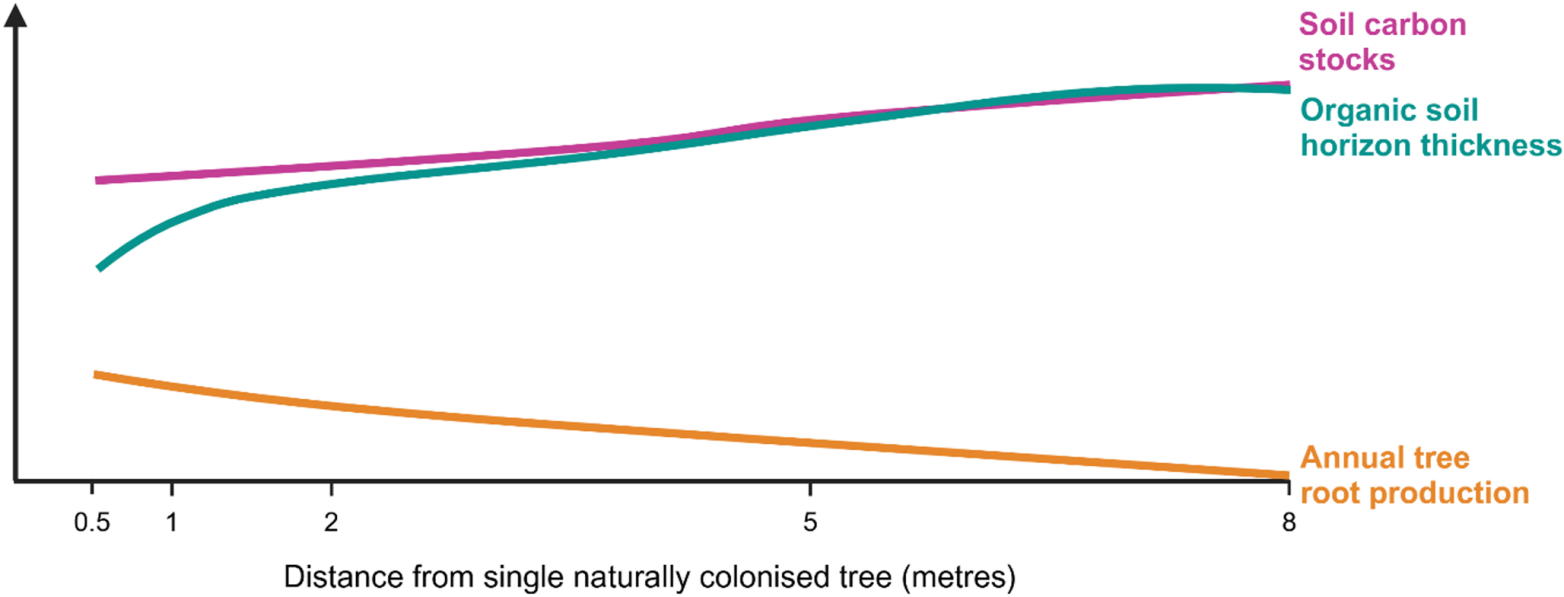
Soil carbon stocks, organic soil horizon thickness, and annual tree root production with distance from single, c. 25-year-old, naturally colonised trees on organo-mineral soils. Based on data from: Housego et al. (2025)

We previously identified that sparse (43 trees ha^− 1^), c. 25-year-old natural colonisation of C-rich organo-mineral soils in the Scottish uplands was associated with soil C losses via reduced soil organic horizon thickness in the major rooting zone of trees (Housego et al. 2025; Fig. 1). However, it is not known whether sparse natural colonisation also alters soil physicochemical properties. The loss of soil C stocks in the organic horizon following natural colonisation could be associated with changes to soil nutrient availability, decomposition rates, pH, and moisture.

Existing studies have examined the effects of dense birch colonisation or planting − 5600-23,000 trees ha^− 1^ in Mitchell et al. (2007), and 650 − 11,000 trees ha^− 1^ in Miles (1981) − onto C-rich soils, also in the Scottish uplands (Miles 1981; Mitchell et al. 2007). In Scotland, where this and the previous studies were conducted, tree planting densities are typically 1600 trees ha^− 1^ for broadleaf forests, and 2500 ha^− 1^ for conifer forests (Scottish Forestry 2021). The aforementioned existing studies found that dense tree establishment was associated with decreased soil moisture, increased N mineralisation, increased decomposition rates, and increased pH compared to adjacent plots of heather moorland (Miles 1981; Mitchell et al. 2007).

**Fig. 2:**
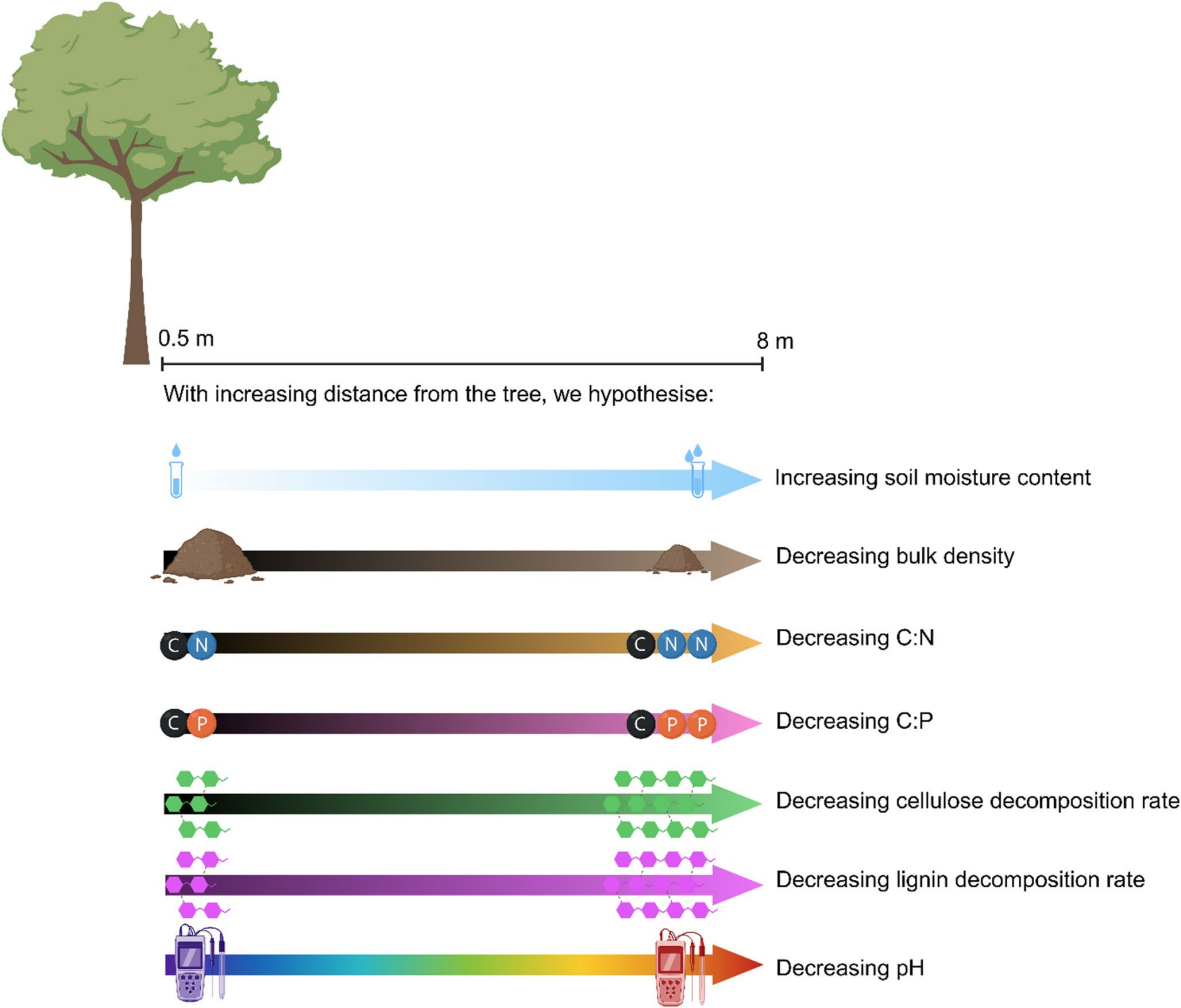
A graphical summary of hypothesised differences in physicochemical properties explored in this study. It shows, with distance from the tree (0–8 m), soil moisture content increasing, and bulk density, C:N, C:P, cellulose decomposition rate, lignin decomposition rate, and pH decreasing. Created in Biorender.com

Should soil physicochemistry also be altered following sparse natural colonisation of organo-mineral soils, this could affect the provision of ecosystem services such as biodiversity and flood alleviation (Hudson 1994; Ilek et al. 2017). Therefore, we investigated the soil physicochemical properties associated with sparse natural colonisation of organo-mineral soils. We employed transects at sites of c. 25-year-old Scots pine or birch natural colonisation in the Cairngorms, Scotland, where soil C stocks had previously been measured at the same points along the transects (Housego et al. 2025). We measured soil physicochemical properties at increasing distances from single, naturally colonised trees, which was also a gradient of increasing soil C stocks and organic horizon thickness. We hypothesised that, with increasing distance along the transects away from the single, naturally colonised trees into open heather moorland, in the organic soil horizon, there would be (1) increased soil moisture, (2) decreased bulk density, (3) decreased C: N and C: P ratios, (4) decreased cellulose and lignin decomposition rates, and (5) decreased pH (Fig. 2).

## Materials and methods

### Sites selection

**Fig. 3:**
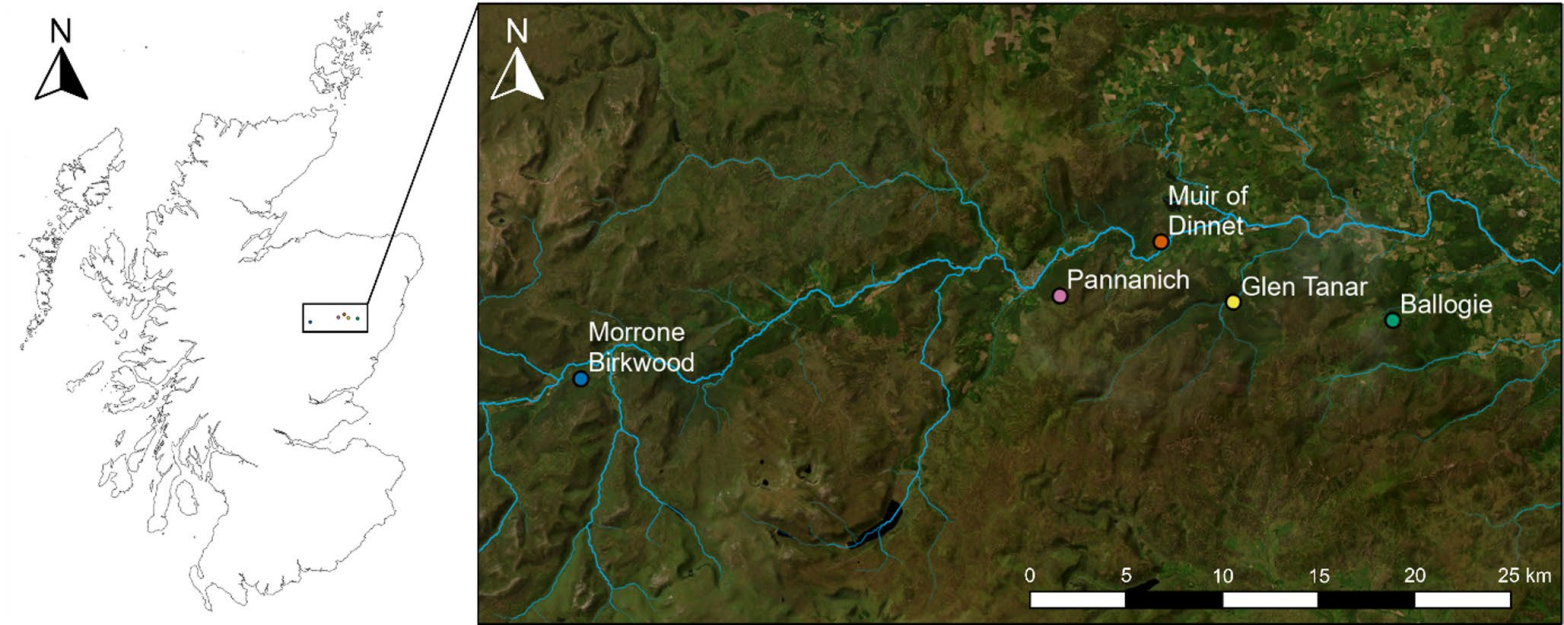
Map of sites. The location of sites within Scotland (left) and Deeside (inset). Maps produced using ArcGIS software by Esri. Basemaps: World Imagery - Esri, Maxar, Earthstar Geographics, and the GIS User Community (Esri et al. 2009); UK Rivers – David Morgan (Morgan 2021). Reproduced from Housego et al. (2025)

Five sites located in Deeside, Aberdeenshire, north-east Scotland, UK were selected (Fig. 3; Table S1; Fig. S1). Each site had c. 25-year-old natural colonisation of either Scots pine, *Pinus sylvestris* L. (Ballogie, Glen Tanar, and Pannanich) or birch, *Betula* spp. (Morrone Birkwood, and Muir of Dinnet) onto *Calluna vulgaris* (L.) Hull-dominated (heather) moorland with organo-mineral soils. Tree ages and establishment method were ascertained from discussions with land managers, historic aerial photographs, and measurements of tree physiology such as height, girth, and branching patterns. All sites fell within the UK National Vegetation Classification H12 *Calluna vulgaris – Vaccinum myrtillus* heath community, and H12b *Vaccinium vitis-idaea – Cladonia impexa* sub-community (Rodwell 1992). All sites were historically managed for deer stalking or grouse shooting, but are now managed for natural colonisation following reductions in deer numbers, although they remain unfenced. Soils were free-draining organo-mineral soils (Table S1) (Housego et al. 2025), i.e. someriumbric podzols (IUSS 2022).

## Sampling design

Single, isolated, naturally colonised trees were identified for sampling. Ten trees were selected at each of the three Scots pine sites, and 15 trees at each of the two birch sites. An 8 m transect was laid from the base of each tree into the heather moorland, ensuring that there were no other trees within 8 m of each sampling point along the transect. Therefore, tree density was 43 trees ha^− 1^. Exploratory sampling prior to sample collection indicated that there were few, if any, tree roots beyond 5 m distance from the tree, which was supported by subsequent sampling of annual root production. Sampling took place at 0.5, 1, 2, 5 and 8 m along the transect. Transects were laid in a range of compass directions and slopes (Housego et al. 2025). The trees at 0 m along the transects represented colonised soils, and the open heather moorland at 8 m along the transects represented uncolonised soils. Increasing distance along the transects, i.e. from 0.5 m to 8 m, therefore represented decreasing tree influence on the soils. Thus, these transects formed a space-for-time substitution that was not confounded by differing land use histories.

## Soil measurements

Soils for bulk density, pH, C:N, and C: P determination were sampled in July-August 2022. Soils were sampled as cores collected using a 5 × 5 cm box corer (Cuttle and Malcolm 1979). The advanced organic (O_a_) soil horizon (IUSS 2022) was identified and partitioned from the other horizons in the field. Three cores were collected per sampling point, with one centred on the sampling point on the transect, and the other two 50 cm perpendicular from this. The thickness of each O_a_ horizon was measured, then the O_a_ horizons at each sampling point bulked for analysis. Soils were initially dried at 30 °C, passed through a 2 mm sieve to remove stones and roots > 2 mm, then a sub-sample was dried at 105 °C for moisture content determination (Housego et al. 2025).

To determine pH, 0.25 g sub-sample was added to 0.01 M calcium chloride (CaCl_2_) in a 1:6 ratio, shaken, and pH measured (Orion Star A211, Thermo Scientific, Loughborough, UK). CaCl_2_ was preferred as a solution because it produces more consistent pH measurements than water (Kissel et al. 2009).

To determine C: N and C: P ratios, a sub-sample was ball milled at 300 revolutions per minute for 10 min (PM 100, Retsch, Haan, Germany). 3.5 mg of the sub-sample was packaged into a tin capsule in a dust-free environment, and %C and %N was determined via elemental analysis (Flash Smart 2000, Thermo Fisher, Waltham, USA) with atropine standards. 100 mg of the sub-sample was digested in 2 ml of 95% H_2_SO_4_ and 2 ml of 30% H_2_O_2_, heated at 120 °C for two hours, then filtered through 25 μm cellulose filter. The total P concentration of the resulting filtrate was then measured via continuous flow analysis (AA3, Seal, Wrexham, UK).

Soil moisture was measured non-destructively as millivolts (mV) using a portable soil moisture probe (ML3 ThetaKit, Delta-T Devices). Soil moisture was measured at three time points, April, July, and September 2023, with three replicates per sampling point per time point. When the final non-destructive measurements were made in September 2023, soil moisture was also measured destructively. A ~ 10–30 g sample of the O_a_ horizon was collected, weighed, dried at 105 °C for 24 h, and weighed again to determine volumetric water content (Gardner 1965).

To measure cellulose decomposition rate, four 55 mm Whatman no.1 filter papers were folded in half, weighed, and placed inside a PVC-coated fiberglass 2 mm mesh pouch sealed with polyester thread. The pouches were installed on the surface of the soil in April 2023, and retrieved in September 2023. To measure lignin decomposition rate, one 115 × 9 mm wooden craft stick was weighed, installed vertically 11.5 cm into the soil in September 2022, and retrieved in September 2023. After retrieval, papers and sticks were air-dried at 30 °C until dry, cleaned, and weighed. Decomposition rate was determined as mass loss per day. Where unable to recover the entire stick, the recovered section was scanned to determine its area as a proportion of its starting area, and mass loss per day was calculated with the starting mass adjusted to the proportion of the stick area recovered.

### Data analysis

All analyses were performed using R version 4.3.1 (R Core Team 2024). Prior to constructing models, pH measurements were converted to [H+] (Krause, 1978). Soil moisture measurements collected using the portable soil probe were converted from mV to percent volumetric water content using a conversion equation for organic soils provided by the manufacturer (Delta-T Devices Ltd, 2017) and the mean for each sampling point calculated. The effect of distance from a single naturally colonised tree (referred to as ‘distance’) on C: N, C: P, cellulose decomposition rate, and lignin decomposition rate, soil pH, and soil moisture were explored using generalised additive mixed models (GAMMs; Table S2), via the R package mgcv (Wood 2011). As Scots pine and birch were sampled at different sites, tree species was not included in the models, as tree species effects could not be separated from site effects. Thus, tree species effects and site effects might be confounded. Site and transect were included as nested random effects. Following inspection of residuals versus fitted plots, where necessary, response variables were transformed to ensure homoscedastic distribution of the errors (Table S2). Spatial autocorrelation of the errors was accounted for by plotting a semi-variogram of the spatial error structure, constructing models with five common correlation structures (exponential, Gaussian, linear, ratio, and spherical), and selecting the model with the lowest Akaike Information Criterion (Pinheiro et al., 2023).

## Results

Distance from single, c. 25-year-old, naturally colonised native trees was associated with increased soil moisture and decreased C: N and C: P, but not associated with altered bulk density, cellulose and lignin decomposition rates, nor soil pH in the O_a_ horizon of organo-mineral soils.

**Fig. 4:**
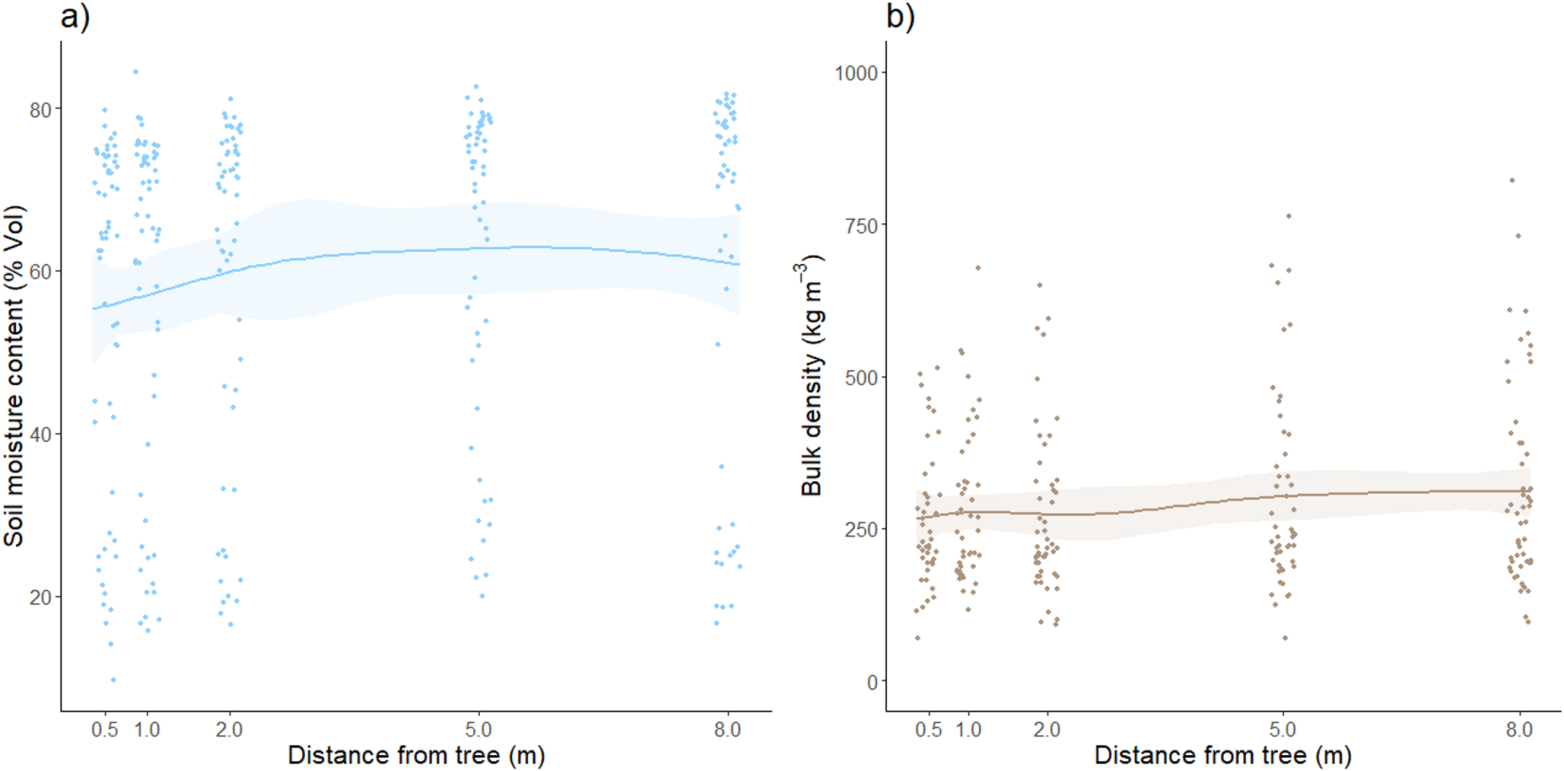
Moisture content and bulk density of the O_a_ soil horizon. (**a**) Soil moisture content measured destructively and (**b**) bulk density of the advanced organic (O_a_) soil horizon with distance from a single naturally colonised tree (*n* = 60). Data is shown with random noise (jitter) on the x-axes to avoid overlapping data points. Shaded areas show ± one standard error around each GAMM smooth regression curve

Soil moisture increased with distance from the tree, (edf = 0.83, *F* = 4.74, *P* = 0.02; Fig. 4a), increasing 11% from 55.2%Vol (SE: ± 2.9%Vol) at 0.5 m, to 61.0%Vol (SE: ± 3.19%Vol) at 8 m (mean ± one standard error presented). This trend was supported by non-destructive measurements of soil moisture taken at three time points throughout the year (*P* < 0.001; Fig. S2; Table S3). However, this did not coincide with differences in bulk density with distance (edf = 1.00, *F* = 2.58, *P* = 0.11; Fig. 4b).

**Fig. 5:**
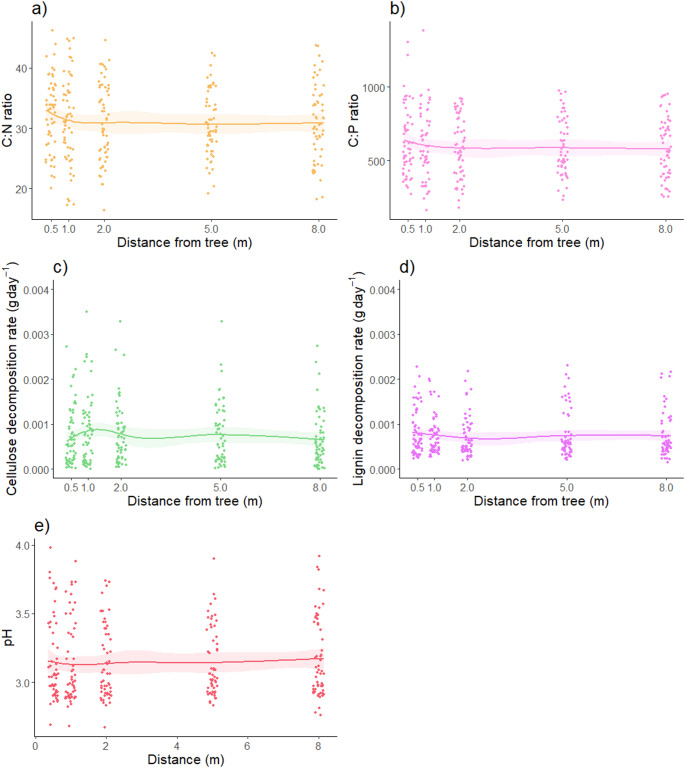
Soil physicochemical properties of the O_a_ soil horizon.** a-b**) Ratio of carbon to nitrogen (**a**) and phosphorus (**b**),** c-d**) decomposition rates of cellulose (**c**) and lignin (**d**), and (**e**) pH in calcium chloride (CaCl_2_) of the advanced organic (O_a_) soil horizon with distance from a single naturally colonised tree (*n* = 60). Data is shown with random noise (jitter) on the x-axes to avoid overlapping data points. Shaded areas show ± one standard error around each GAMM smooth regression curve

The C: N and C: P of the O_a_ horizon decreased (Fig. 5a-b) with distance from the tree. C: N decreased 5% from 32.4 (SE: ± 0.84) at 0.5 m, to 30.9 (SE: ± 0.84) at 8 m, while C: P decreased 8% from 631 (SE: ± 31.2) at 0.5 m, to 581 (SE: ± 26.6) at 8 m. Only the decrease in C: P was statistically significant (edf = 0.76, *F* = 3.20, *P* = 0.04), but the decrease in C: N was only narrowly non-significant (edf = 0.69, *F* = 2.25, *P* = 0.07). Neither the decomposition rates of cellulose nor lignin (Fig. 5c-d) varied significantly with distance from the tree (edf < 0.001, *F* = 0, *P* = 0.51, and edf = < 0.001, *F* = 0, *P* = 0.76, respectively). Likewise, O_a_ horizon pH (Fig. 5e) did not vary significantly with distance from the tree (edf = 0.63, *F* = 1.74, *P* = 0.10).

## Discussion

This study showed that sparse natural tree colonisation was associated with decreased soil moisture, and increased C: N and C: P, but contrary to other studies (Miles 1981; Mitchell et al. 2007), it was not associated with changes in bulk density, cellulose and lignin decomposition rates, or soil pH. These changes in soil moisture and C: N and C: P ratios coincided with the soil C losses associated with decreased O_a_ horizon thickness and increased tree root production observed by Housego et al. (2025). Differences in soil physicochemical properties, if present, were localised to the major rooting zone of the tree, which was within 2 m distance from the base of the trees (Fig. 1).

Soil moisture of the O_a_ horizon was 11% lower in the major rooting zone of trees compared to 8 m along the transect in open heather moorland, which supported our first hypothesis. Natural colonisation can decrease soil moisture (Miles 1981; Mitchell et al. 2007), as the canopy interception and transpiration rates of trees can be greater than that of the existing vegetation community (Rutter 1966; Soulsby et al. 2017). The greater ventilation associated with sparse natural colonisation can increase interception rates relative to denser tree cover, which might further decrease soil moisture (Haria and Price 2000). When tree species such as Scots pine and birch colonise ecosystems, they can drive the fungal community to become more ectomycorrhizal-dominated (Clemmensen et al. 2015), which might also contribute to the lower soil moisture associated with natural colonisation. Ectomycorrhizal fungi (EcM) could be increasing tree water uptake and transpiration rates relative to heather moorland vegetation, as some ectomycorrhizal fungi form aggregations of hyphae similar to plant roots (‘rhizomorphs’) that transport water from the soil to their tree hosts (Koide and Wu 2003).

Our second hypothesis was not supported, as bulk density did not vary with distance from the tree. When peatlands are drained and planted with trees, soil moisture loss reduces support for the soil structure, causing it to collapse, and the organic horizon shrinks and increases in density (Cannell et al. 1993; Minkkinen and Laine 1998). This can be further exacerbated by the mass of the growing trees exerting additional pressure on the soil (Minkkinen and Laine 1998). However, we found no support for this during early colonisation of organo-mineral soils, where tree density and biomass were low and no drainage had taken place.

Soil C: N and C: P ratios were higher in the major rooting zone of the trees compared to in open heather moorland, supporting our third hypothesis. Given that O_a_ horizon %C did not differ along the transects despite decreased O_a_ horizon C stocks (Housego et al. 2025), greater C: N and C: P ratios indicate lower soil N and P stocks. Thus, tree colonisation was associated with lower N stocks despite Scots pine and birch litters typically containing more N and P than existing heather moorland vegetation community, both in terms of concentration and mass (van Meeteren et al. 2007). This could be because trees extract and uptake N from the soil at a greater rate than the heather moorland vegetation community, for example via ‘N-mining’ (Hicks et al. 2020; Clemmensen et al. 2021). Tree colonisation of heather moorland soils is associated with the fungal community composition shifting to become more EcM-dominated. Although EcM fungi degrade soil organic matter though extracellular enzyme production (Zak et al. 2019), they extract N at higher rates than the C mineralisation that they drive, resulting in high soil C: N (Clemmensen et al. 2021). EcM might also provide access to soil P (Plassard et al. 2011; Cairney 2011), which is the next most limiting soil nutrient in heather moorland after N (Kirkham 2001), but this is less well-studied than EcM N-extraction.

Our fourth hypothesis was not supported, as we did not detect decreases in cellulose and lignin (major components of tree litter) decomposition rates along the transects. This was unexpected, as decomposition rates are typically linked to soil C: N and C: P. Litter composition varied along the transect, with the volume of tree litter decreasing with distance from the trees (Housego et al. 2025). Changes in litter volume and composition following tree establishment typically promote increased decomposition, as Scots pine and birch litter is decomposed faster than heather litter (Parker et al. 2025). In turn, C, N, and P are released from the litter into the soil at greater rates (Finzi et al. 1998; van Meeteren et al. 2007). Labile dissolved fractions of C, N, and P, resulting from litter decomposition, can further drive microbial activity, and therefore further the breakdown of old soil organic C (Moscatelli et al. 2005). However, litter decomposition can be slowed by lower soil moisture and lower N and P stocks (van Meeteren et al. 2007), so the observed decreases in soil moisture and C: N and C: P ratios in the major rooting zone of trees might have counteracted increased decomposition rates following natural colonisation. However, the 55% soil moisture of the soils in the major rooting zones of trees were still relatively moist; further research is required to determine whether these levels of soil moisture would limit decomposition relative the 61% soil moisture of uncolonised soils. 25 years after sparse colonisation might also be too soon for the soil microbial community composition to shift, and thus for decomposition processes to shift, in response to the altered litter composition and quality. Indeed, it can take 40–60 years for soil fungal taxa richness to peak in second rotation forest with an ericaceous understory (Li et al. 2020). This might be even longer for sparse colonisation, where tree-associated fungi are not already present in the soil (Collier and Bidartondo 2009).

Previous studies detected differences in decomposition rates in soils beneath trees and heather moorland using similar methods to those employed in this study (e.g. Mitchell et al. 2007, 2021), and the 2 mm mesh size of the pouches encasing the papers should not have prevented access by decomposers (Bradford et al. 2008; Bokhorst and Wardle 2013). Upon collection, the papers and sticks were visibly decomposed, and if left for another season, would have been irretrievable (pers. obs.). However, unlike the previous studies, which installed the papers within the soil organic horizon, in this study, the papers were installed on the soil surface instead, to better represent where tree litterfall is first deposited. Furthermore, replication at each sampling point might have been insufficient (Karberg et al. 2008) given the low tree densities, and the spatial heterogeneity of soil C (Vanguelova et al. 2016) that results in a spatially heterogenous soil community and decomposition processes (Nunan et al. 2020). In Housego et al. (2025), soil C stocks were sampled with a 5 × 5 cm box corer three times at each sampling point, meaning that 75.0 cm^− 2^ of soil surface area was sampled, compared to the 23.8 cm^− 2^ and 12.8 cm^− 2^ surface area of the papers and sticks, respectively, and the overall number of replicates (transects) was 60. In the study by Mitchell et al. (2007) that detected significant increases in decomposition rates between dense birch planting and heather moorland control plots in Scotland, the surface areas of the sticks and papers and the number of days for which they were installed were similar, and replication at each sampling point was the same. However, the overall number of replicates (plots) was higher, at 216. The density of trees was also greater (40,000 trees ha^− 1^, versus 43 ha^− 1^ in our study), thus the likelihood of tree-associated decomposition processes interacting directly with the sticks and papers was greater (Mitchell et al. 2007).

Our fifth hypothesis, that soil pH would decrease with distance from the trees, was also not supported. Soil pH is determined by the amounts of different elements in the soil, so increases in litter volume and C and N content (Finzi et al. 1998; Vesterdal et al. 2013) following colonisation of heather moorland was anticipated to increase soil pH. However, soil pH did not vary with distance from the trees, despite C: N decreasing with distance from the trees. In turn, increased soil pH mediated by tree litter inputs can increase decomposition rates, as less acidic soils are more favourable to earthworms, which mix organic matter between the organic and mineral horizons (Finzi et al. 1998), thus also driving thinner O_a_ horizons and soil C losses (Miles 1981). Soil pH might also increase following colonisation as trees take up different amounts of elements from the soil than the existing vegetation community (Finzi et al. 1998; van Meeteren et al. 2007). However, soil pH changes are not always detected at the 25-year timescale of our study. This could be due to the inherent pH buffering capacity of organo-mineral soils (Sollins et al. 1996), such as those at our study sites. Indeed, increased soil pH was observed 25 years after birch colonisation and over 50 years after Scots pine colonisation of lowland mineral heather moorland soils (Mitchell 1997), and 50 years after birch planting onto organo-mineral heather moorland soils (Miles 1981). But 23 years after birch planting onto organo-mineral heather moorland soils (Mitchell et al. 2007), and 25–50 years after Scots pine colonisation of lowland mineral heather moorland soils, no effect on soil pH was found (Mitchell 1997). Instead, Mitchell et al. (2007) found that tree size and tree density were greater drivers of soil pH increases following birch colonisation of heather moorland (Mitchell et al. 2007), saying “old but small trees had little effect [on soil physicochemical properties]” (p.550). Given that the trees in our study were small (mean diameter at breast height = 11.6 cm) and colonisation was sparse, it is also possible that they would not alter soil pH.

## Conclusions

Sparse natural colonisation of organo-mineral soils was associated with decreased soil moisture and increased C: N and C: P, but not with any changes to bulk density, decomposition, or pH, in the O_a_ horizon. Indeed, denser tree establishment appears to have a greater effect on soil physicochemical properties (Miles 1981; Mitchell et al. 2007). The sparsity of trees in our study might explain why we did not detect differences in decomposition and pH following natural colonisation, whereas Mitchell et al. (2007) and Miles (1981) did at sites with higher biomass, denser birch establishment of a similar age. Where soil physicochemical properties varied with distance from the trees into open heather moorland, differences were localised to within 2 m of the tree, thus implying minimal impacts on soil physicochemical properties at the ecosystem scale in early, sparse colonisation. These localised differences might affect soil biodiversity and hydrology in patches around the trees. For example, the less moist soils under colonised trees are associated with increased species abundance and richness of soil fauna such as mites, springtails and nematodes, and with a more bacteria-dominated soil microbial community compared to the moister soils in open heather moorland (Mitchell et al. 2007; Brooker et al. 2008). Early natural colonisation can also contribute to flood alleviation (van Meerveld et al. 2021). At what density trees begin to alter the soil physicochemical properties of the entire ecosystem is unknown, but might coincide with overlapping rooting zones and/or canopy closure. Further work is required to determine this; for example, via a chronosequence approach whereby the approaches used in this study are repeated at sites in Deeside Valley with different densities of natural colonisation, or via existing thinning experiments, similar to those of Miles and Mitchell et al. at sites across Scotland and northern England (Miles 1981; Mitchell et al. 2007). The effects of sparse colonisation on soil physicochemical properties might also increase over time, for example with cumulative tree litter inputs, regardless of whether density increases or not. This could be explored by repeating this study at the same sites in another 25 years, where tree densities are unlikely to substantially increase unless management approaches change to reduce grazing pressures.

## Data Availability

The datasets and code generated and analysed during the current study are available in the Zenodo repository: 10.5281/zenodo.14289726.
